# Reductions in Resting Blood Pressure in Young Adults When Isometric Exercise Is Performed Whilst Walking

**DOI:** 10.1155/2017/7123834

**Published:** 2017-05-07

**Authors:** Anthony W. Baross, David A. Hodgson, Sarah L. Padfield, Ian L. Swaine

**Affiliations:** ^1^Sport and Exercise Science, University of Northampton, Northampton, UK; ^2^Sports Science, University of Greenwich, Chatham Maritime, UK

## Abstract

Aerobic and isometric training have been shown to reduce resting blood pressure, but simultaneous aerobic and isometric training have not been studied. The purpose of this study was to compare the changes in resting systolic (SBP), diastolic (DBP), and mean arterial blood pressure (MAP) after 6 weeks of either (i) simultaneous walking and isometric handgrip exercise (WHG), (ii) walking (WLK), (iii) isometric handgrip exercise (IHG), or control (CON). Forty-eight healthy sedentary participants (age 20.7 ± 1.7 yrs, mass 67.2 ± 10.2 kg, height 176.7 ± 1.2 cm, male *n* = 26, and female *n* = 22) were randomly allocated, to one of four groups (*n* = 12 in each). Training was performed 4 × week^−1^ and involved either treadmill walking for 30 minutes (WLK), handgrip exercise 3 × 10 s at 20% MVC (IHG), or both performed simultaneously (WHG). Resting SBP, DBP, and MAP were recorded at rest, before and after the 6-week study period. Reductions in resting blood pressure were significantly greater in the simultaneous walking and handgrip group than any other group. These results show that simultaneous walking and handgrip training may have summative effects on reductions in resting blood pressure.

## 1. Introduction

Hypertension has been identified as a major health risk and one of the most prevalent risk factors for cardiovascular disease (CVD) and all-cause mortality [1, 2]. It can be defined as a mean resting systolic blood pressure of ≥140 mmHg and/or a mean resting diastolic pressure of ≥90 mmHg [3, 4]. It is projected that with every 2 mmHg increase in systolic blood pressure, regardless of the initial blood pressure level, there is an associated 7–10% increased risk of ischemic heart disease or stroke. Estimates of the global prevalence of hypertension are thought to be around one billion [5], with around a third of the European adult population suffering from hypertension. Increased physical activity has been reported as an important low-cost intervention for reducing the incidence, modifying the associated risks, and for use in the treatment of hypertension [5–7].

Several meta-analyses have identified that various types of training can lower resting blood pressure [3, 7–11]. Aerobic training has been reported to lower resting systolic blood pressure (SBP; −5 to −7 mmHg) and diastolic blood pressure (DBP; −2 to −7 mmHg) [7, 8, 12]. Resistance training has been shown to induce similar reductions [3]. One particular form of resistance training (isometric training) has been shown to be especially potent in reducing resting blood pressure (relative to the amount of training performed). Previous research has identified a significant decrease in resting SBP (−5 to −13 mmHg) and DBP (−3 to −9 mmHg) following either upper [13, 14] or lower body [15, 16] isometric training.

Few studies have investigated the effects of combined exercise protocols on resting blood pressure [17–19]. The results from these studies are equivocal. Two studies reported no significant changes in resting blood pressure following combined aerobic and resistance training [17, 18]. In contrast, Sigal et al. [18] identified small nonsignificant reductions in SBP and DBP in aerobic training and combined training groups. Calders et al. [19] recently identified significant decreases in SBP and DBP in combined and aerobic training groups compared to a control group. Importantly, the reductions in blood pressure were significantly greater in the combined training group (SBP = 15 mmHg; DBP = 2 mmHg) compared to the aerobic training group (SBP = 11 mmHg; DBP = no change).

In these studies the two component modes of exercise were combined in a sequential way, where participants undertook one element of the training programme followed by the other (e.g., resistance training followed by a 30-minute treadmill walk). However, there have not been any studies of the effects on resting blood pressure of combined simultaneous training involving aerobic exercise (e.g., walking) whilst performing isometric exercise (e.g., handgrip). Since there are simple, easy-to-use handgrip devices it is possible to perform this type of exercise whilst walking. This simultaneous approach would be advantageous because the time spent in training would be reduced compared to a sequential protocol. Therefore, the purpose of this study was to compare the effects on resting systolic blood pressure (SBP), diastolic blood pressure (DBP), and mean arterial pressure (MAP) of 6 weeks of training, involving either (i) simultaneous walking and isometric handgrip (WHG), (ii) walking only (WLK), (iii) isometric handgrip only (IHG), or (iv) control conditions (CON).

## 2. Method

### 2.1. Participants

A total of 48 healthy sedentary participants, who were university students (male *n* = 26 and female *n* = 22) were randomly allocated, to four groups which undertook six weeks of either walking training (WLK; *n* = 12, female, *n* = 6, mean ± SD: age 20.7 ± 1.6 yrs, mass 64.0 ± 8.9 kg, height 172.2 ± 4.2 cm, and SBP 126.7 ± 3.7 bpm), isometric handgrip training (IHG; *n* = 12, female, *n* = 6, mean ± SD: age 20.9 ± 2.0 yrs, mass 66.9 ± 8.8 kg, height 176.6 ± 6.7 cm, and SBP 127.1 ± 4.0 bpm), simultaneous walking and handgrip training (WHG; *n* = 12, female, *n* = 5, mean ± SD: age 20.0 ± 0.5 yrs, mass 68.8 ± 10.4 kg, height 181.3 ± 6.9 cm, and SBP 127.8 ± 4.5 bpm), or acting as a control group (CON; *n* = 12, female, *n* = 5, mean ± SD: age 21.3 ± 2.0 yrs, mass 69.2 ± 12.5 kg, height 177.4 ± 7.0 cm, and SBP 127.9 ± 4.2 bpm). All university students were eligible for participation. However, volunteers were excluded if they reported any recent (6 months) history of medical treatment for serious illness such as high blood pressure, orthopaedic injury, viral illness, or surgical procedure. Also, habitually active students were excluded. Volunteers had to be able to confirm that they had not participated in regular exercise training (3 or more times per week) for 12 months prior to enrolment in our study. All baseline and posttraining measures in women were taken during the same phase of the menstrual cycle. The CON group maintained their normal daily routine during the 6-week intervention period. After receiving institutional ethical approval each participant received a detailed information sheet explaining the experimental protocol and potential risks involved and then signed and completed an informed consent form and pretest medical screening questionnaire.

### 2.2. Procedure

#### 2.2.1. Baseline Measures

On the first of two initial visits to the laboratory participants were familiarised with the equipment and test procedures. During the second visit the resting baseline measures (heart rate and blood pressure) were recorded using a heart rate monitor (Polar Beat, Polar Electro, Kempele, Finland) and an automatic blood pressure monitor (UA-767 Plus, A&D Company, Ltd., Tokyo, Japan). Resting heart rate (HR) and blood pressure (BP, SBP, DBP, and MAP) were recorded following 15-minute rest, with the participants in the supine position. Three measurements were taken at 1-minute intervals and the average value was used to determine resting HR and BP [20].

This BP monitor has been reported to provide accurate measurement of systolic and diastolic blood pressure compared to mercury sphygmomanometry (SBP, −0.93 ± 5.1 mmHg; DBP, −0.41 ± 4.73 mmHg [21]). However, the reproducibility of blood pressure measurement for this specific automated device has not been established. Several other automated devices, similar to the one used in the present study, have been assessed for reproducibility [22]. Automated devices are generally not as reproducible as mercury sphygmomanometry and have been shown to exhibit somewhat greater variation in repeated measurement values. Approximately 60% of measurements exhibited less than 5 mmHg difference when using automated devices (whereas 80% of repeated measurements for mercury sphygmomanometry show less than 5 mmHg difference [22]).

In our study, the BP measures were subsequently recorded again, following the 6-week training intervention. Following measurement of resting BP each participant's isometric handgrip maximum voluntary contraction (MVC) was determined for both the left and right hand, using a handgrip dynamometer (Zona Plus Hand Grip device, Zona Health B. V., Netherlands). The participants training torque was calculated as 20% of the recorded MVC. The 6-week training intervention was selected because although many aerobic training programmes are around 12 weeks, many isometric resistance training programmes have been much shorter (4 weeks [23]). Therefore, we selected a 6-week programme to reflect the combination of aerobic and isometric training. We also used a lower isometric resistance training intensity than previously used, to take account of the fact that our participant groups include sedentary men and women.

#### 2.2.2. Training

Following the initial baseline measures the WLK group undertook 30 minutes of treadmill walking at an exercise intensity of 6.5 km·hr^−1^, on four days a week for 6 weeks, using a mains powered treadmill (GX100, Powerjog, Birmingham, UK). The WHG group completed the same walking protocol with the addition of 10 s of isometric handgrip exercise at 20% of their MVC, three times during the 30-minute walk (5 minutes, 15 minutes, and 25 minutes). The three bouts of handgrip exercise were performed using each hand alternately (2 of the 3 bouts with the dominant hand). The IHG group completed the same isometric exercise training protocol (standing) without the treadmill walk (squeezing the handgrip device at 5, 15, and 25 minutes). All training sessions were performed at the University of Northampton in a consistent laboratory environment under supervision.

#### 2.2.3. Data Analysis

The data were assessed for normal distribution and for parametric assumptions [24]. Statistical analysis was performed using Microsoft Excel, SPSS 20. Our participant group was a fairly homogeneous group (young, healthy university students) and therefore all groups were fairly closely matched in relation to age and BMI. Furthermore, due to the randomised nature of the allocation to groups it was assumed that there would be small, nonsignificant, differences between group baseline measures for age, BMI, smoking, and initial level of fitness. However, it has been reported (Millar et al., 2007) that the magnitude of the blood pressure reductions is related to the initial resting values. Therefore, repeated measures analysis of covariance (RM ANCOVA) was used to determine the effects of the training interventions on resting blood pressure and to compare the group changes (delta) in resting blood pressure, using the baseline values as the covariate. Post hoc analysis (Bonferroni) was then used to further determine specific significant differences between groups. An alpha level of 0.05 was accepted as being significant.

## 3. Results

### 3.1. Subject Characteristics

Baseline data (Table 1) indicated that there were no significant differences between groups (WHG versus WLK versus IHG versus CON) for body mass, resting heart rate (RHR), resting systolic blood pressure (RSBP), resting diastolic blood pressure (RDBP) and resting mean arterial pressure (RMAP) (*P* > 0.05 in all cases).

### 3.2. Effects of Training on Resting Blood Pressure

#### 3.2.1. Resting Systolic Blood Pressure

**Figure 1:**
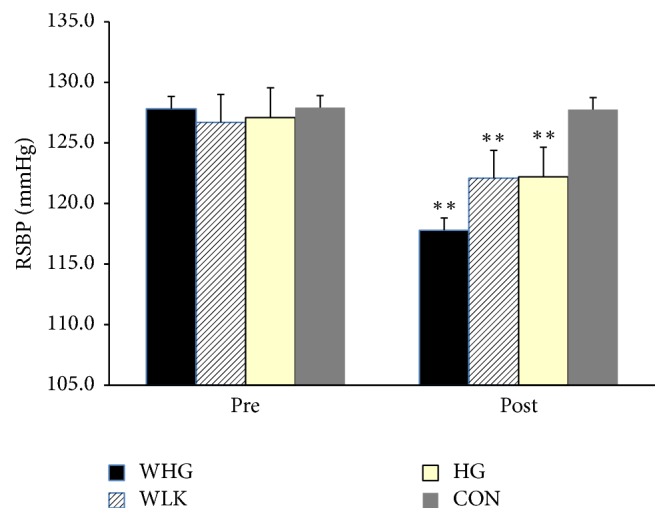
Resting systolic blood pressure (RSBP) for simultaneous walking and handgrip (WHG), walking (WLK), handgrip (HG), and control (CON) groups before and after training. ^*∗∗*^*P* value < 0.01.

Resting SBP was reduced significantly after 6 weeks of simultaneous walking and handgrip training (WHG, 127.8 ± 4.5 mmHg to 117.8 ± 3.6 mmHg) versus the CON group (127.9 ± 4.3 mmHg to 127.8 ± 4.3 mmHg) (*P* < 0.001, Figure 1). The reductions in RSBP after training were not significant in either the WLK (126.7 ± 3.4 mmHg to 122.1 ± 4.2 mmHg) or the IHG group (127.1 ± 4.0 mmHg to 122.2 ± 3.7 mmHg) compared to the CON group (*P* > 0.05 in both cases).

#### 3.2.2. Resting Diastolic Blood Pressure

Although there was a slight decrease in RDBP for all training groups (WHG, 77.2 ± 2.8 mmHg to 73.5 ± 3.8 mmHg; WLK, 77.7 ± 3.0 mmHg to 75.9 ± 2.4 mmHg; IHG, 76.3 ± 3.3 mmHg to 73.9 ± 3.9 mmHg) these decreases were not significant compared to the CON group (77.0 ± 1.8 mmHg to 76.5 ± 3.0 mmHg; *P* > 0.05 in all cases).

#### 3.2.3. Resting Mean Arterial Blood Pressure (RMAP)

**Figure 2:**
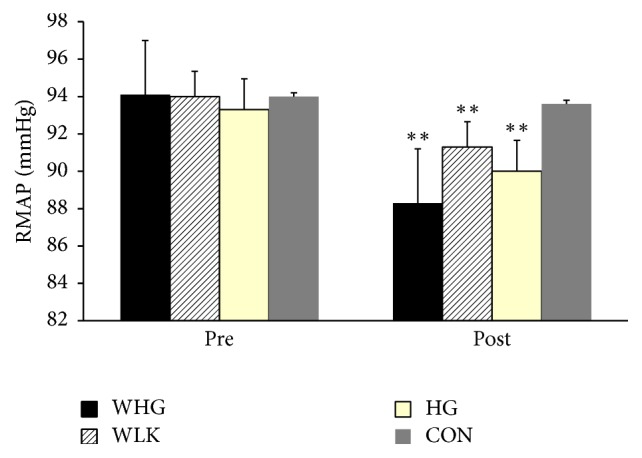
Resting mean arterial pressure (RMAP) for the simultaneous walking and handgrip (WHG), walking (WLK), handgrip (HG), and control (CON) groups before and after training. ^*∗∗*^*P* value < 0.01.

Analysis of the RMAP showed a significant decrease of 5.8 mmHg in the WHG training group (94.1 ± 2.7 mmHg to 88.3 ± 3.1 mmHg) relative to the CON group (94.0 ± 2.2 mmHg to 93.6 ± 3.1 mmHg) (*P* < 0.001). Both the WLK and IHG groups RMAP showed a small decrease (WLK, 94.0 ± 3.0 mmHg to 91.3 ± 2.3 mmHg; HG, 93.3 ± 3.1 mmHg to 90.0 ± 2.8 mmHg). However, neither of these group decreases was significant relative to the CON group (94.0 ± 2.2 mmHg to 93.6 ± 3.1 mmHg) as shown in Figure 2 (*P* > 0.05 in both cases).

#### 3.2.4. Magnitude of the Changes in Blood Pressure

Additional analysis of the differences in the reductions in RSBP (Δ SBP) following the 6-week training programme showed that there was a significant main effect (*P* < 0.001) and a significant group × time interaction for WHG, WLK, and IHG (*P* < 0.001 in all cases). Post hoc analysis of the Δ SBP values for the WHG group (10.1 ± 3.1 mmHg) showed that Δ SBP was greater in this training group compared to the other two training groups (IHG and WLK) and compared to the CON group (*P* < 0.001 in all cases). The difference in Δ SBP in the remaining two training groups WLK and IHG were 4.6 ± 2.5 mmHg and 4.9 ± 2.6 mmHg, respectively. These reductions were significantly less than the WHG group but significantly greater than the CON group (*P* < 0.001 in both cases) but not significantly different from each other (*P* > 0.05).

## 4. Discussion

The main finding of the present investigation was that performing isometric handgrip exercise whilst walking caused a reduction in resting systolic blood pressure, which far exceeded the reductions seen after walking or isometric handgrip only. The significant reductions in resting SBP following the completion of all three training programmes (WHG, −10.1 mmHg; WLK, −4.6 mmHg; IHG, −4.9 mmHg) and the magnitude of the reduction in RMAP (Δ MAP; WHG, −5.8 mmHg; WLK, −2.7 mmHg; IHG, −3.3 mmHg) were comparable or higher than that seen in previous isometric-only studies or sequential combined training studies [14, 17–19, 25, 26]. The benefits of such reductions are evident in the current literature, which suggests that decreases in blood pressure of this magnitude would provide significant public health benefits [27]. A reduction of 2 mmHg has been associated with a 7% reduction in mortality from cardiovascular disease, a 10% decrease in mortality resulting from strokes [28], and a 3% reduction in all-cause mortality [29].

The reduction in resting blood pressure observed in the walking group was similar to previous investigations in younger adults (SBP, −5 to −2; DBP, −3 to −1 [12, 25, 30, 31]), although generally the length of the training interventions (8–25 weeks) and/or the training intensities (60–86% VO_2 max_ ) in those previous studies were greater than those of the present study [25, 30, 31]. A few studies (Palmer, 1995) [31] have reported similar reductions in SBP (−5 to −2.8) to those observed in our study, with similar training volume and intensity. However, those studies used older participants (Palmer, 1995) [31] and premenopausal women (Palmer, 1995) or reported higher baseline resting SBP [31].

Research exploring the effects of isometric handgrip training on the resting blood pressure of younger normotensive adults has also produced similar results (SBP, −4 to −5 mmHg [14, 26]). However, both of these studies incorporated a greater number of exercise bouts (4 repetitions) of longer duration (2 min). These studies also used a higher exercise intensity equivalent to 30% MVC in addition to longer (8 weeks) training interventions [14, 26]. However, our walking and isometric exercise group showed changes that were greater than many of the previous isometric resistance training studies. The reasons for this are, at present, difficult to explain. Our participants were sedentary (no regular exercise training for 12 months prior to enrolment in our study) and this might have meant that their initial level of fitness was particularly low. Indeed, it may be worthwhile to explore the impact that initial level of fitness has, on the effects of this type of training, in future research.

It is difficult to explain why the small amount of isometric handgrip training in the present study should elicit significant reductions in SBP (3 × 10 s at 20% MVC). There is some evidence that brief, and low-intensity, isometric training programmes can induce reductions in resting blood pressure. For example, Devereux et al. [16] reported significant reductions in SBP (−5 mmHg) and MAP (−3 mmHg) following only 4 weeks of isometric bilateral-leg training at an exercise intensity that equated to approximately 20% MVC. Baross et al. [32] reported significant reductions in SBP (−11 mmHg) and MAP (−5 mmHg) following isometric bilateral-leg training at relatively low intensities (approximately 14% MVC). However, the studies of Devereux et al. [16] and Baross et al. [32] used much longer periods of exercise, than we used in the present study; therefore the training stimulus is likely to have been much greater. In the present study training was performed 4 times per week, whereas most previous isometric training studies have used 3 times per week, and this may have contributed to our significant findings.

To the authors' knowledge no other studies have investigated the resting blood pressure lowering effects of simultaneous combined walking and isometric exercise (handgrip) training in young normotensive adults. The present study is therefore the first to show that 6 weeks of simultaneous treadmill walking and isometric handgrip training, 4 times per week, significantly lowers RSBP and RMAP compared to walking or isometric handgrip only training programmes of the same duration and frequency. Due to the novelty of the training intervention it is difficult to compare the WHG results to previous studies. However, the present findings can be compared to previous sequential combined resistance and aerobic exercise training studies [17–19]. Calders et al. [19] reported significant changes in resting SBP (−15 mmHg) in their combined training group which is similar to the −10 mmHg reported here. The duration of our training programme and the exercise intensity were considerably higher in the Calders et al. [19] study. Their combined training group was also older (mean 42 yrs) and hypertensive (SBP, 140 mmHg) at baseline. Of course, it is necessary to demonstrate the effect of simultaneous walking and isometric exercise, in a much larger group of participants, before our finding can be adopted as a clinically meaningful intervention. Furthermore, many researchers believe that it is necessary to demonstrate the blood pressure lowering effects of exercise training interventions on ambulatory blood pressure, in addition to resting blood pressure, before definitive conclusions about efficacy can be drawn [33].

### 4.1. Possible Mechanisms

It is important to discuss whether these findings inform understanding of the mechanism responsible for the training-induced reductions in resting blood pressure. Of course, we did not directly study any measures that are thought to relate to the mechanisms. Nevertheless, the findings of the present study suggest that the effects of walking and the effects of isometric training on resting blood pressure could be summative. This idea has been suggested previously by Millar et al. (2012). In their “letter to the editor,” these authors suggested that, because they had observed significant reductions in resting blood pressure after isometric training, even when participants were already aerobically trained, this could suggest independent blood pressure lowering mechanisms for aerobic and isometric training. Otherwise, in a key meta-analysis by Cornelissen and Fagard [9] it was suggested that a decrease in the activity of the autonomic nervous system was most likely involved in the training-induced reduction of blood pressure and systemic vascular resistance, as evidenced by the consistently sizeable reductions (29%) in plasma norepinephrine levels in fitter compared with untrained participants. It was also suggested that the 20% decrease of plasma renin activity supports the involvement of the renin-angiotensin system [34, 35] possibly via a reduction in the activity of the sympathetic nervous system [36]. If this were indeed the mechanism involved, it would infer that the autonomic nervous system effects of the two exercise modalities were summative when performed simultaneously.

## 5. Conclusions

The findings of this study provide new evidence that performing isometric handgrip whilst walking can cause significantly greater reductions in resting blood pressure than either walking or isometric handgrip training. Furthermore, if brief periods of isometric exercise, when performed simultaneously whilst walking, are shown to be effective in larger samples, it might be possible to develop simple alternative methods for performing this type of exercise, without the need for specialist equipment. Doing so could make this type of exercise much more accessible to a greater proportion of the population. This could increase the potentially beneficial effects of these types of exercise on resting blood pressure. The sizeable reduction in resting blood pressure emphasises the antihypertensive potential of simultaneous handgrip and walking training especially since the effects were evident in the present study in young individuals whose resting blood pressure was considered to be normal. Since it is believed that the relationship between blood pressure and the risk of cardiovascular disease has no lower threshold, the reductions identified in this study in normotensive individuals may still have clinical benefits [37]. These findings also give support to the suggestion of Millar et al. (2012) that the effects of each type of exercise on resting blood pressure might be summative when performed simultaneously.

## Figures and Tables

**Table 1:** Control and trained groups resting baseline data.

	CON	WHG	WLK	HG
Age (yrs)	21.3 ± 2.0	20.0 ± 0.5	20.9 ± 2.0	20.7 ± 1.6
Body mass (Kg)	69.2 ± 12.5	68.8 ± 10.4	64.0 ± 8.9	66.9 ± 8.8
Height (m)	177.4 ± 7.0	181.3 ± 6.9	172.2 ± 4.2	176.6 ± 6.7
BMI (kg/m^2^)	22.1 ± 0.8	21.0 ± 0.7	21.7 ± 0.6	21.6 ± 0.6
RHR (bpm)	67.4 ± 2.7	67.8 ± 1.7	66.0 ± 3.1	65.5 ± 3.1
RSBP (mmHg)	127.9 ± 4.2	127.8 ± 4.5	126.7 ± 3.7	127.1 ± 4.0
RDBP (mmHg)	77.0 ± 1.8	77.2 ± 2.8	77.7 ± 3.0	76.3 ± 3.3
RMAP (mmHg)	94.0 ± 2.2	94.1 ± 2.7	94.0 ± 3.0	93.3 ± 3.1

Values are means ± SD (CON group *n* = 12; WHG group *n* = 12; WLK group *n* = 12; HG group *n* = 12). BMI, body mass index; RHR, resting heart rate; RSBP, resting systolic blood pressure; RDBP, resting diastolic blood pressure; RMAP, resting mean arterial pressure.
